# Which Is the Wiser?

**Published:** 1873-02

**Authors:** 


### WHICH IS THE WISER?

I HAVE two friends whose names are, or might be, Smith and Brown. Their habits of life and the results these habits produce are worthy of being recorded; they may serve as mirrors in which some other folks may see themselves as others see them. They are both somewhat over thirty years of age, are both married and have several children, and are comfortably well off for this world's goods. Smith is a good, sensible fellow, of more than average culture, whose opinion or advice on most matters I would as soon ask as I would that of any man I know. His wife is a right clever, good-natured, hard-working woman—somewhat given to the fripperies of fashion, but always conscientiously doing, as she believes, everything she can to promote the well-being of her husband and family. Neither Smith, who has been a great reader, nor his wife, who has not, had ever paid the least attention to hygienic matters. They both "came up" in families where the largest liberty to do as they pleased was given to the children, and they have continued the same course with their own, trusting in Providence, as they said, to carry them safely through. They have had five children, two of them are already in their graves, and some one of the others, or one of the parents, is needing the doctor's care continually—their door being one of the regular stands at which his patients go to look for his gig. No one who knows their habits of life needs to have studied the great problem—*how to secure good health*—any great length of time, not to know that there are good reasons, which ought to be patent to these parents, why sickness and death and the long train of anxieties and heart-rendings which they bring with them have invaded their house. Let us see, then, if *we* can find a sufficient cause. These parents are fond of what they call good-living. In the selection of food for their table, the gratification of the appetite is the only point consulted, and their table is always bountifully supplied with the richest and most appetizing food—if such rubbish may be called food. Their children are allowed to come to the table at which their parents eat as soon as they can sit up alone in a chair, are always asked what they would like to eat, and their every wish in this particular is gratified. I dined there one day when two of the children were at the table—one three and the other five years old, perhaps—and I saw that they ate heartily of roast pork with plenty of gravy, and finished with mince pie, and this at six o'clock in the afternoon! At another time I saw the mother come in, a few minutes before dinner, with a paper of candies, eating of them herself, and dealing them out liberally to her children!

These instances only serve to show the ordinary habits of eating in the family, and are in no wise unusual for them. Another matter. In the winter these children are kept housed almost the whole time—sometimes not going out of the house from one week's end to another, for fear the poor little things would take cold; and of course they had colds nearly the whole time. The natural consequence of such management of children, as any one who has paid any attention to the study of health knows very well, is certain sickness and probable early death. A death, which is the result of the ignorance or indifference of the parents to hygienic laws, is but very little short of homicide; and the time will come, we predict, when it will be generally so regarded. In a family where such habits of living prevail, it is no wonder the doctor reaps a rich harvest; no wonder the husband's yearly balance-sheet makes so poor a showing; no wonder that the father and mother suffer from care and anxiety—their hearts lacerated with grief; and no wonder that they are always bewailing their misfortunes, and that they experience so much of that side of life which they call *dark*. It *is* a wonder, however, that any clergyman can be found so profoundly ignorant as to slander his Maker by exclaiming, as one did in the course of his remarks at the funeral of one of these children, "God has called this little one away." I ached to "speak in meeting" and tell the reverend gentleman that God had done no such thing; that it was not His will that any child should die thus early, and that it was only in consequence of disobeying His laws that death had come to the poor little creature before him. This child, who had, as I had already learned, been ailing for several days, had been allowed to come to the table at dinner late in the day and eat heartily of lobster-salad and drink freely of milk, because she asked for these things, and her mother's only thought was to gratify the child's every wish. The consequence was, as might be expected, that about midnight the child had a violent attack of *cholera morbus*, and within twenty-four hours was a corpse! In what sense could it be said then, pray, that "God called the poor little thing away?" It was a cheap platitude for the clergyman to utter, and perhaps a solace to the wounded hearts of its parents; but it was a slander on Divine Wisdom, nevertheless.

II.

Brown and his wife and Smith and his wife were married about the same time. Brown, intellectually, was far below Smith. His reading had been confined more to the practical side of life, and somehow it happened that, while yet a youth, the importance of the subject of health had attracted his attention, and he studied it thoroughly, and had so far indoctrinated his wife before her marriage with the subject, that they were already fully agreed that good health for themselves and offspring, if they should have any, was to be the foundation-stone of their domestic happiness. They provided themselves with the best books on the Management of Infancy, on Hygiene and on Physiology; studied them and profited by what they learned. They found out how best to care for their offspring before birth, and how to treat them after. They knew what was the proper food for them, and this they provided and saw that it was good and properly cooked. The children understood that their parents knew what was the proper food to give them to eat, as well as the proper quantity, and they also knew that they were certain to be helped to it at the proper time, and they were never asked what they would like to eat. They came to the table with their parents, ate what was given them, and always seemed contented and happy. These children were encouraged also to take robust exercise out of doors, always when the weather was fair, and very often when it was not;. cold and wet had little fear for them. When the weather was bad they dressed warmer. I have often seen them of a cold winter's day come into the house, after a brisk play out of doors, with complexions which, for beauty and healthful appearance, would attract the attention of the most indifferent observer, and I very rarely heard of their having colds.

Well, Brown's children are all alive and well to-day; all hearty and robust. Aside from the bills which have been rendered to him for obstetrical services, ten dollars will pay all his family's doctor's bills for thirteen years. The saving in money, however, is trifling compared with the exemption from the anxiety and grief which the Smiths suffer.

There is not a reader of this journal, probably, who cannot put his finger upon a dozen families within the range of his acquaintance who are living like Smith's, but I very much fear he will not know one like Brown's. These sketches are from life, however, and their truthfulness will be readily recognized by the parties themselves and their friends. The moral to be drawn is manifest and need not be put in words.

Now, young man—you I mean, whose name I saw mentioned in the papers this morning, as about to marry the wealthy Miss ——, of Thirty-fourth street, whose *trousseau* was said to have been ordered from Paris at a cost of ten thousand dollars—let me whisper in your ear, that ten dollars judiciously invested in good books which will teach you and your wife how to live, so as to enjoy good health yourselves, and to secure the same for your children, will yield you and your little wife infinitely more happiness—provided you are willing to profit by the teaching—than any amount of money spent on the wedding *trousseau*. Hold up before you, then, these two pictures of Smith and Brown and decide for yourself, WHICH IS THE WISER ?

